# The role of glucose disposal efficiency in predicting stroke among older adults: a cohort study

**DOI:** 10.3389/fneur.2025.1540160

**Published:** 2025-03-11

**Authors:** Zongren Zhao, Yu Liu, Jinyu Zheng, Jing Li

**Affiliations:** Department of Neurosurgery, Affiliated Huaian Hospital of Xuzhou Medical University, Huaian, China

**Keywords:** eGDR, CHARLS, stroke, subgroups, interquartile range

## Abstract

**Background:**

Glucose disposal rate (eGDR) has recently been validated as a surrogate marker of insulin resistance, providing a novel approach to assess metabolic health. However, the relationship between changes in eGDR levels and stroke incidence remains underexplored. The current study aims to investigate the impact of eGDR control on stroke incidence and related events.

**Methods:**

Data were obtained from the China Longitudinal Study on Health and Retirement (CHARLS). The analysis included 6,375 participants aged 45 and above with complete stroke and eGDR data from the CHARLS for 2011, 2013, and 2015. Logistic multivariable regression examined the relationship between eGDR and stroke, using threshold analysis to identify inflection points. we categorized participants into distinct subgroups based on sociodemographic variables to see the relationship between stroke and other variables.

**Results:**

Out of the 8,060 individuals analyzed in the cohort, 821 were diagnosed with new-onset stroke. There was a notable negative correlation found between new-onset risk of stroke and eGDR, with each Interquartile Range (IQR) increment in eGDR leading to a 38% risk reduction [OR: 0.62; 95% CI: (0.45,0.84)]. Stratified analyses revealed age as a potential modifier in the age-stroke relationship (*P* for interaction = 0.01).

**Conclusion:**

Poorly controlled eGDR level is associated with an increased risk of stroke in middle-aged and elderly people. Monitoring changes in eGDR may help identify individuals at high risk of stroke early.

## 1 Introduction

Stroke is a critical public health issue worldwide, causing significant disability and mortality. It was responsible for disability-adjusted life years (DALYs) and deaths globally (1, 2). Although high-income countries have seen a decline in stroke-related DALYs and fatality rates due to advancements in treatment and rehabilitation, low- and lower-middle-income countries face an increasing prevalence of stroke (3, 4). This highlights the need for affordable and practical methods to identify individuals at high risk of developing stroke.

Insulin resistance (IR), characterized by the body’s reduced response to insulin (5, 6), has been identified as a contributor to stroke risk, primarily through its role in promoting atherosclerosis (7). The hyperinsulinemic-euglycemic clamp test is considered the most precise method for assessing insulin sensitivity, but its complexity and high cost limit its routine use in clinical practice (8). Consequently, alternative, less invasive approaches such as the triglyceride-glucose index, homeostasis model assessment-IR (9), and estimated glucose disposal rate (eGDR) have gained attention (10). Among these, eGDR offers a straightforward and accessible evaluation method using hypertension status, waist circumference, and glycated hemoglobin A (HbA1c) levels as indicators (11).

Studies have shown that eGDR is an effective tool for assessing insulin resistance and is associated with higher mortality risk in diabetic patients (12). Moreover, its predictive value in cardiovascular diseases has been investigated. For instance, a large-scale study involving over 100,000 individuals with type 2 diabetes found that those with lower eGDR levels had a reduced stroke risk (13). Another follow-up study reported that a one-standard-deviation increase in eGDR was linked to a lower risk of cerebrovascular disease (14), suggesting that eGDR may serve as an independent risk marker for cardiovascular events.

However, most research to date has focused on static eGDR measurements, and little is known about how changes in eGDR over time relate to stroke incidence (15). Additionally, the variability in blood glucose levels and glycemic control across populations further complicates the picture. To address these gaps, our study utilizes data from the China Health and Retirement Longitudinal Study (CHARLS) to explore the relationship between dynamic changes in eGDR and stroke risk, aiming to provide new insights into its potential as a predictive tool for identifying individuals at risk of stroke.

## 2 Method

### 2.1 Data source and participants

This study leveraged data from the China Health and Retirement Longitudinal Study (CHARLS), a nationwide survey targeting individuals aged 45 and older in China. CHARLS aims to provide a comprehensive understanding of aging’s socioeconomic and health impacts by collecting extensive information on physical and mental health, socioeconomic status, demographic characteristics, and social networks. The survey consistently achieves a high response rate, exceeding 80% in annual interviews.

The dataset used for this analysis was publicly accessible and obtained with approval from institutional review boards. CHARLS received ethical clearance from the Biomedical Ethics Committee of Peking University (IRB00001052-11015), and all participants provided written informed consent. The study followed the Strengthening the Reporting of Observational Studies in Epidemiology (STROBE) guidelines.

For this 10-year longitudinal analysis (2011–2020), data from 2011 served as the baseline. Participants were excluded if they had incomplete age data, were younger than 45, lacked eGDR information, or had a baseline diagnosis of stroke. Among the initial cohort of 17,707 individuals, 8,060 participants were included in the final analysis. Of these, 7,239 individuals did not experience a stroke during the follow-up period, while 821 cases of new-onset stroke were recorded.

### 2.2 Data collection

Data were gathered through structured household interviews conducted by trained interviewers. The interviews collected demographic details, such as age, gender, marital status, education level, geographic location, and history of chronic illnesses. These variables were crucial for adjusting the analysis to account for confounding factors that could influence the relationship between eGDR and stroke. eGDR = 21.158–0.09 * WC (waist circumference) - 3.407 * Hypertension - 0.551*HbA1c.

Additionally, information on health behaviors—including smoking status, alcohol consumption, and body mass index (BMI)—was collected. BMI was measured using standardized protocols, while data on smoking and drinking were self-reported. Including these variables provided a more holistic understanding of factors influencing stroke risk and eGDR. The CHARLS data collection adhered to ethical guidelines established by the Peking University Research Ethics Committee, ensuring participant confidentiality and autonomy throughout the study.

### 2.3 Statistical analysis

Continuous variables were summarized as mean ± standard deviation (SD) or medians, while categorical variables were expressed as percentages. The primary focus of the analysis was to examine the relationship between the interquartile range of eGDR (eGDR_IQR) and the risk of new-onset stroke.

To explore this association, multivariate logistic regression models were applied. Odds ratios (ORs) with 95% confidence intervals (CIs) for eGDR_IQR were calculated across three models:

Model 1: A crude model with no adjustments. Model 2: Adjusted for sociodemographic factors, including age, gender, marital status, education, BMI, and geographic location. Model 3: Further adjusted for additional potential confounders, such as smoking status, alcohol consumption, hypertension, and diabetes.

Interaction analyses were conducted to assess whether sociodemographic or health factors moderated the association between eGDR and stroke risk. The dose–response relationship between eGDR and stroke risk was visualized using restricted cubic splines, with key percentiles (5th, 35th, 65th, and 95th) as reference points.

To evaluate the incremental predictive value of eGDR_IQR beyond traditional clinical markers, it was incorporated into a baseline logistic regression model. All analyses were conducted using R software (version 4.3.0). R package called FearsR. Download link: devtools::install_github(‘charlszj/chrhelp’);chrhelp::install_charlsR(‘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’).

## 3 Results

### 3.1 Study population characteristics

Table 1 presents the demographic and clinical characteristics of the study cohort, which included 8,060 participants, 821 of whom were newly diagnosed with stroke. The median age of the cohort was 58 years, with 3,647 males (45.25%) and 4,413 females (54.75%). Comparative analysis showed that individuals with new-onset stroke tended to have lower educational attainment and were less likely to be smokers compared to those without stroke (*p* < 0.05). Additionally, a significant difference in eGDR was observed, with stroke patients exhibiting a shorter eGDR (9.47 vs. 8.35, *p* < 0.0001).

**Table 1:** Baseline characteristics of study population by stroke status at follow-up.

Variable	Total (*n* = 8,060)	No (*n* = 7,239)	Yes (*n* = 821)	Statistic	*p* value
eGDR	9.35 ± 2.29	9.47 ± 2.26	8.35 ± 2.32	13.14	**<0.0001**
Age	58.72 ± 8.92	58.45 ± 8.91	61.08 ± 8.69	−8.18	**<0.0001**
Sex				1.07	0.30
Female	4,413 (54.75)	3,978 (54.95)	435 (52.98)		
Male	3,647 (45.25)	3,261 (45.05)	386 (47.02)		
Marital_status				3.51	0.06
Married	7,171 (88.97)	6,457 (89.20)	714 (86.97)		
Non-married	889 (11.03)	782 (10.80)	107 (13.03)		
Education				7.47	**0.02**
College and higher	89 (1.10)	85 (1.17)	4 (0.49)		
Elementary school and below	5,655 (70.16)	5,050 (69.76)	605 (73.69)		
High school	2,316 (28.73)	2,104 (29.06)	212 (25.82)		
Location				0.01	0.92
Rural	5,398 (66.97)	4,850 (67.00)	548 (66.75)		
Urban	2,662 (33.03)	2,389 (33.00)	273 (33.25)		
Smoke				8.43	**0.01**
Current smoker	2,411 (29.91)	2,158 (29.81)	253 (30.82)		
Former smoker	659 (8.18)	572 (7.90)	87 (10.60)		
Never	4,990 (61.91)	4,509 (62.29)	481 (58.59)		
Drink				0.44	0.51
No	5,420 (67.25)	4,859 (67.12)	561 (68.33)		
Yes	2,640 (32.75)	2,380 (32.88)	260 (31.67)		
Hypertension				134.07	**<0.0001**
No	4,908 (60.89)	4,562 (63.02)	346 (42.14)		
Yes	3,152 (39.11)	2,677 (36.98)	475 (57.86)		
DM				33.77	**<0.0001**
No	6,925 (85.92)	6,275 (86.68)	650 (79.17)		
Yes	1,135 (14.08)	964 (13.32)	171 (20.83)		
BMI				40.39	**<0.0001**
Normal	5,528 (68.59)	5,044 (69.68)	484 (58.95)		
Obesity	394 (4.89)	336 (4.64)	58 (7.06)		
Overweight	2,138 (26.53)	1,859 (25.68)	279 (33.98)		
Dyslipidemia				44.37	**<0.0001**
No	4,869 (60.41)	4,462 (61.64)	407 (49.57)		
Yes	3,191 (39.59)	2,777 (38.36)	414 (50.43)		
Heart.disease				58.21	**<0.0001**
No	7,175 (89.37)	6,509 (90.26)	666 (81.52)		
Yes	853 (10.63)	702 (9.74)	151 (18.48)		

### 3.2 Longitudinal association between eGDR interquartile range and new-onset stroke risk

**Figure 1:**
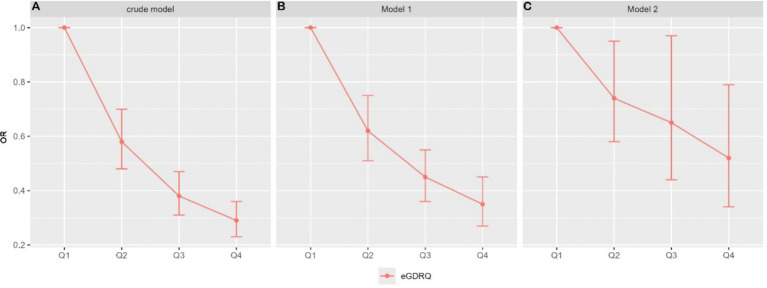
eGDR quartiles and their corresponding odds ratios (OR) for stroke across three models.

Table 2 and Figure 1 summarize the findings from the multivariate regression analyses. The results indicate a significant inverse relationship between eGDR Interquartile Range (eGDR_IQR) and the risk of new-onset stroke when eGDR_IQR is treated as a continuous variable. In the fully adjusted model, each IQR increment in eGDR_IQR was associated with a 14% reduction in stroke risk (OR: 0.864; 95% CI: 0.784–0.954). When eGDR_IQR was categorized into quartiles, the highest quartile (Q4) demonstrated a notable 31% lower risk of stroke onset compared to the lowest quartile (Q1) (OR: 0.69; 95% CI: 0.48–0.96).

**Table 2:** Prospective associations between baseline eGDR with follow-up new-onset stroke in CHARLS.

Character	Crude model		Model 1		Model 2	
	95%CI	*P*	95%CI	*P*	95%CI	*P*
eGDR_IQR	0.44 (0.39,0.50)	<0.0001	0.5 (0.43,0.58)	<0.0001	0.62 (0.45,0.84)	0.002
eGDRQ						
Q1	Ref		Ref		Ref	
Q2	0.58 (0.48,0.70)	<0.0001	0.62 (0.51,0.75)	<0.0001	0.74 (0.58,0.95)	0.02
Q3	0.38 (0.31,0.47)	<0.0001	0.45 (0.36,0.55)	<0.0001	0.65 (0.44,0.97)	0.03
Q4	0.29 (0.23,0.36)	<0.0001	0.35 (0.27,0.45)	<0.0001	0.52 (0.34,0.79)	0.002
*p* for trend		<0.0001		<0.0001		0.003

Crudel model: eGDR_IQR. Model 1: eGDR_IQR, age, sex, location, marital_status, BMI. Model 2: eGDR_IQR, age, sex, location, marital_status, BMI, smoke, drink, hypertension, DM, dyslipidemia, heart.disease.

### 3.3 Association of eGDR quartiles with stroke probability across three models

**Figure 2:**
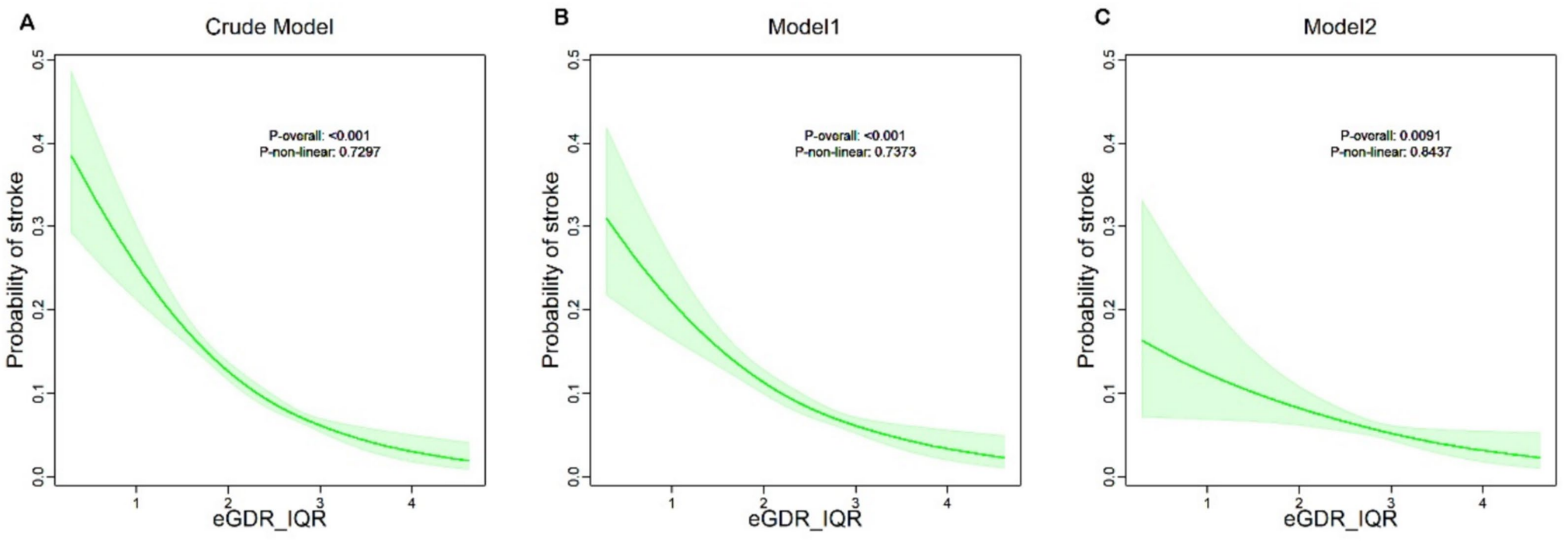
eGDR quartiles and their corresponding stroke probabilities in Crude, Model 1, and Model 2.

Figure 2 provides an in-depth analysis using Restricted Cubic Splines (RCS), illustrating a linear dose–response relationship between eGDR and the risk of new-onset stroke in the fully adjusted model (*P* overall <0.0091, *P* non-linear = 0.8437). Across all three models, a consistent negative correlation was observed, indicating that higher eGDR levels are linked to a reduced likelihood of stroke.

### 3.4 Stratified analysis

**Figure 3:**
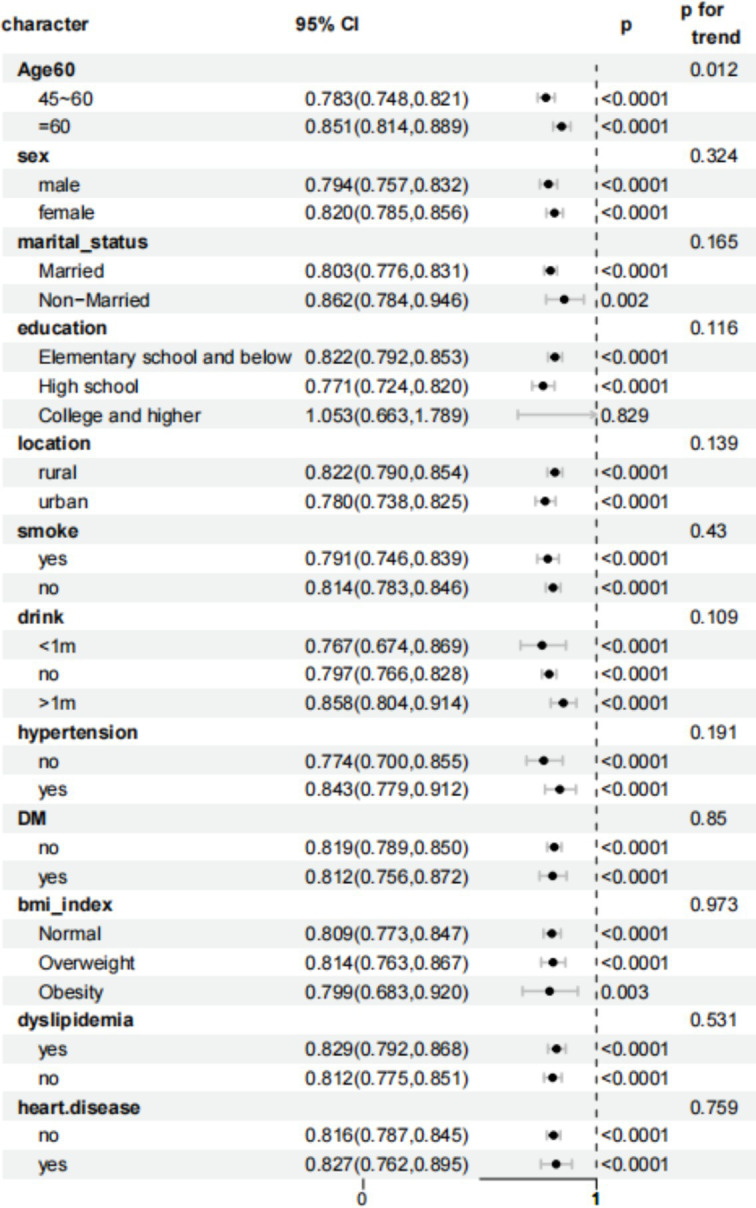
Forest plot of stratified analysis of the association of eGDR_IQR and new-onset risk of stroke.

To assess the robustness of the association between eGDR Interquartile Range and the risk of new-onset stroke, participants were stratified into subgroups based on sociodemographic characteristics and medical history, as shown in Figure 3. Interaction analysis revealed that age moderated this relationship, with a significant interaction effect (*P* for interaction = 0.012). An increase in eGDR by one IQR was linked to a reduced likelihood of stroke across most subgroups. However, no significant association was observed among individuals with a college-level education or higher.

## 4 Discussion

This study investigates the association between the estimated glucose disposal rate (eGDR) and the incidence of stroke in a middle-aged and elderly population. Our findings reveal a significant inverse correlation between eGDR and stroke risk, with each IQR increase in eGDR associated with a 38% reduction in the risk of new-onset stroke. This relationship was consistent across different models, even after controlling for various confounding factors.

The observed inverse relationship between eGDR and stroke risk is consistent with the established role of insulin resistance (IR) in the pathogenesis of cardiovascular diseases, including stroke. IR is known to contribute to the development of atherosclerosis, a major precursor to stroke (16, 17). The eGDR, as a noninvasive measure of IR, provides a practical tool for assessing an individual’s risk of stroke. Moreover, insulin resistance may lead to impaired glucose metabolism and chronic hyperinsulinemia, which can exacerbate vascular damage and promote the formation of thrombi, increasing the likelihood of ischemic stroke. Our results support the use of eGDR as a valuable marker for identifying individuals who may benefit from interventions aimed at improving insulin sensitivity and reducing stroke risk.

The stratified analysis indicated that age may modify the relationship between eGDR_IQR and stroke risk, suggesting that the impact of eGDR on stroke risk may vary across different age groups. This finding underscores the importance of considering age when interpreting eGDR in the context of stroke risk assessment.

The strength of our study lies in its large sample size and the use of a longitudinal design, which allows for the examination of temporal relationships between eGDR and stroke incidence. Additionally, the use of multiple models with progressive adjustments for confounders enhances the reliability of our findings. However, our study is not without limitations. The use of self-reported data for certain variables, such as smoking and drinking habits, could introduce bias. Furthermore, the cross-sectional nature of the eGDR assessment does not allow for the determination of causality (18). Also, our study is the presence of time-dependent bias in the follow-up data. Time-dependent bias can occur when the exposure (eGDR levels) and covariates (such as lifestyle factors and comorbidities) change over time, potentially influencing the outcome (stroke incidence). This bias can affect the validity of our findings. Additionally, our study did not include younger individuals, who may have different risk profiles for stroke and insulin resistance. Future research should aim to validate our findings in more diverse populations.

Despite these limitations, our study’s findings have important implications for clinical practice and public health. Monitoring eGDR could serve as a noninvasive and cost-effective approach to identify individuals at high risk of stroke, particularly in middle-aged and elderly populations. Early identification of at-risk individuals could facilitate the implementation of targeted interventions to improve insulin sensitivity and reduce the burden of stroke.

In conclusion, our study highlights the potential of eGDR as a predictive marker for stroke risk in middle-aged and elderly adults. Future research should focus on validating these findings in other populations and exploring the underlying mechanisms that link eGDR, insulin resistance, and stroke risk.

## 5 Conclusion

This longitudinal cohort study identified a significant association between eGDR and the risk of new-onset stroke in the general population. Our findings highlight the importance of enhancing eGDR levels among middle-aged and older adults as a crucial strategy for the primary prevention of stroke.

## Data Availability

Publicly available datasets were analyzed in this study. This data can be found at: https://charls.charlsdata.com.
